# Lengthened circadian rhythms in mice with self-controlled ambient light intensity

**DOI:** 10.1038/s41598-024-58415-x

**Published:** 2024-04-02

**Authors:** Jun Ogasawara, Nobuyoshi Matsumoto, Yuki Takeuchi, Kotaro Yamashiro, Masato Yasui, Yuji Ikegaya

**Affiliations:** 1 Department of Pharmacology, School of Medicine, Keio University, Tokyo, 160-8582 Japan; 2 Laboratory of Chemical Pharmacology, Graduate School of Pharmaceutical Sciences, The University of Tokyo, 7-3-1 Hongo, Bunkyo-ku, Tokyo, 113-0033 Japan; 3 Institute for AI and Beyond, The University of Tokyo, Tokyo, 113-0033 Japan; 4 Center for Information and Neural Networks, National Institute of Information and Communications Technology, Suita City, Osaka 565-0871 Japan

**Keywords:** Neuroscience, Psychology

## Abstract

Laboratory animals are typically maintained under 12-h light and 12-h dark (12:12 LD) conditions with a daytime light intensity of ~ 200 lx. In this study, we designed an apparatus that allowed mice to self-select the room light intensity by nose poking. We measured the behavioral rhythms of the mice under this self-controlled light regimen. The mice quickly learned the relationship between their nose pokes and the resulting changes in the light intensity. Under these conditions, the mice exhibited free-running circadian behavior with a period of 24.5 ± 0.4 h. This circadian period was ~ 1 h longer than that of the same strain of mice when they were kept in constant darkness (DD) after 12:12 LD entrainment, and the lengthened period lasted for at least 30 days. The rhythm of the light intensity controlled by the mice also exhibited a similar period, but the phase of the illuminance rhythm preceded the phase of the locomotor activity rhythm. Mice that did not have access to the light controller were also entrained to the illuminance cycle produced by the mice that did have access to the light controller, but with a slightly delayed phase. The rhythm was likely controlled by the canonical circadian clock because mice with tau mutations in the circadian clock gene CSNK1E exhibited short periods of circadian rhythm under the same conditions. These results indicate that the free-running period of mice in the wild may differ from what they exhibit if they are attuned by forced light cycles in laboratories because mice in their natural habitats can self-control their exposure to ambient light, similar to our experimental conditions.

## Introduction

Physiological circadian rhythms are attuned to the rhythms of our surrounding environment^1^. Organisms use reliable environmental time cues to entrain their autonomous circadian clocks to the environmental 24-h cycle. Light is the most reliable signal, but in nature, the intensity and spectrum of the light change both over the course of each day and across seasons. Under laboratory conditions, animals are commonly housed under strict 12-h light:12-h dark conditions with a fixed light intensity of ~ 200 lx during the light phase. Since it is uncommon that a den is provided in the home cage in the vivarium, the laboratory mice are exposed to the full brightness of the light during the day. However, this produces an unnatural condition for mice since mice in nature rarely come out of the nest and are exposed to light during the day; in the wild, they bathe in the sunlight of their own will. In this light, we question whether and how circadian rhythms differ between mice in the laboratory and those in the wild.

To address this question, we designed an apparatus that allows mice to freely choose the light intensity in their cage by nose poking and measured their circadian behavioral rhythms under self-controlled light exposure conditions.

## Results

### Self-controlled illumination leads to a free-running period longer than 24 h

**Figure 1:**
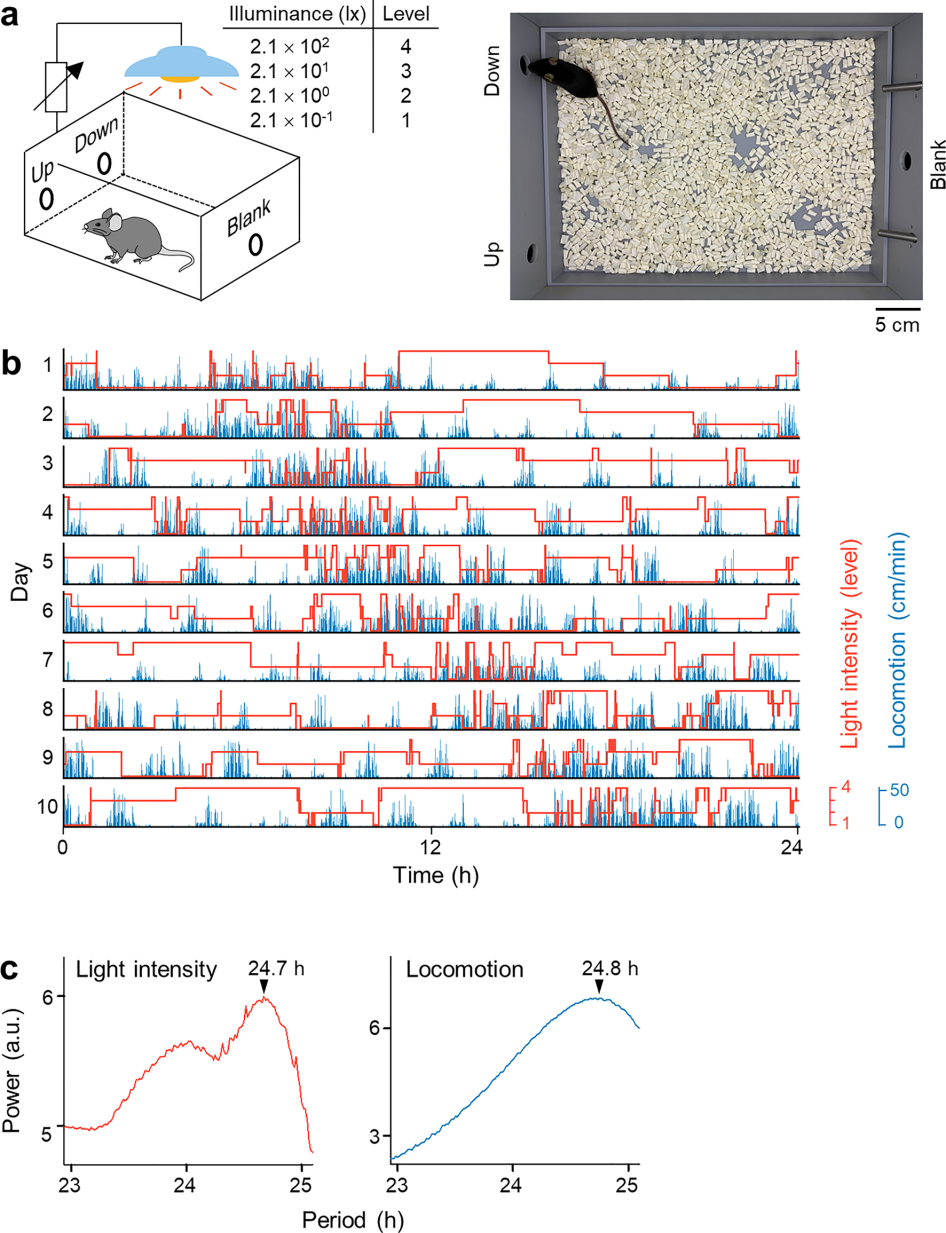
Self-controlled chamber light intensity prolongs the period of the circadian rhythm. (**a**) Schematic (left) and photograph (right) of the experimental chambers. Mice were able to modulate the illuminance of their chambers across four levels by poking their noses into the Up and the Down holes on the wall. The Blank hole was nonfunctional; that is, it did not change the illuminance. (**b**) Actogram of illuminance (red) and locomotion (blue) over 10 d. The horizontal axis represents time, and each row represents a single day. (**c**) Power spectra of the rhythms of illuminance (red) and locomotion (blue) indicate the circadian rhythm. The arrowhead indicates the peak of each cycle.

We designed a chamber with three nose-poke holes in its walls (Fig. 1a, Supplementary Movie). Two of these holes served as switches that adjusted the brightness of the light-emitting diode (LED) in the ceiling of the chamber, and the remaining hole did not have any function (*i.e.*, “Blank”). One of the functional holes (*i.e.*, “Up”) elevated the brightness of the chamber by one step for each nose poke, whereas the other (*i.e.*, “Down”) lowered the chamber brightness by one step for each nose poke. This apparatus had four illuminance levels: 0.21 lx (level 1), 2.1 lx (level 2), 21 lx (level 3), and 210 lx (level 4). A total of 12 chambers were prepared, and each chamber was placed in a single soundproof box in a quiet (< 40 dB) underground room without exterior windows. Unless otherwise specified, a mouse was kept in a single chamber for 10 days (d), during which time the experimenters did not approach the room, and the behaviors of the animals were remotely monitored via an overhead camera.

Upon being housed in this chamber, the mice quickly learned to control the light intensity of the chamber. Under self-controlled lighting conditions, the mice exhibited a free-running circadian behavioral period longer than 24 h (Fig. 1b). No significant differences were observed in the amount of time spent under each of the four illuminance levels over 10 d of the experiment (Supplementary Fig. 1a; 27 ± 13% (level 1), 26 ± 9% (level 2), 25 ± 9% (level 3), and 23 ± 11% (level 4); χ^2^ = 0.21, *P* = 0.65, *n* = 76 mice, Cochran-Armitage trend test). The mean illuminance during the 10-d experiment was 47.4 ± 85.3 lx. Consistent with the nocturnal behavior of mice in the wild, our mice were more active under lower illuminance levels (Supplementary Fig. 1b; 6.8 ± 3.4 cm/min (level 1), 6.6 ± 3.0 cm/min (level 2), 5.9 ± 2.9 cm/min (level 3), 5.2 ± 2.5 cm/min (level 4); *P* = 1.5 × 10^–4^, *JT* = 13,830, *Z* = -3.62, *n* = 76, Jonckheere-Terpstra test^2^).

**Figure 2:**
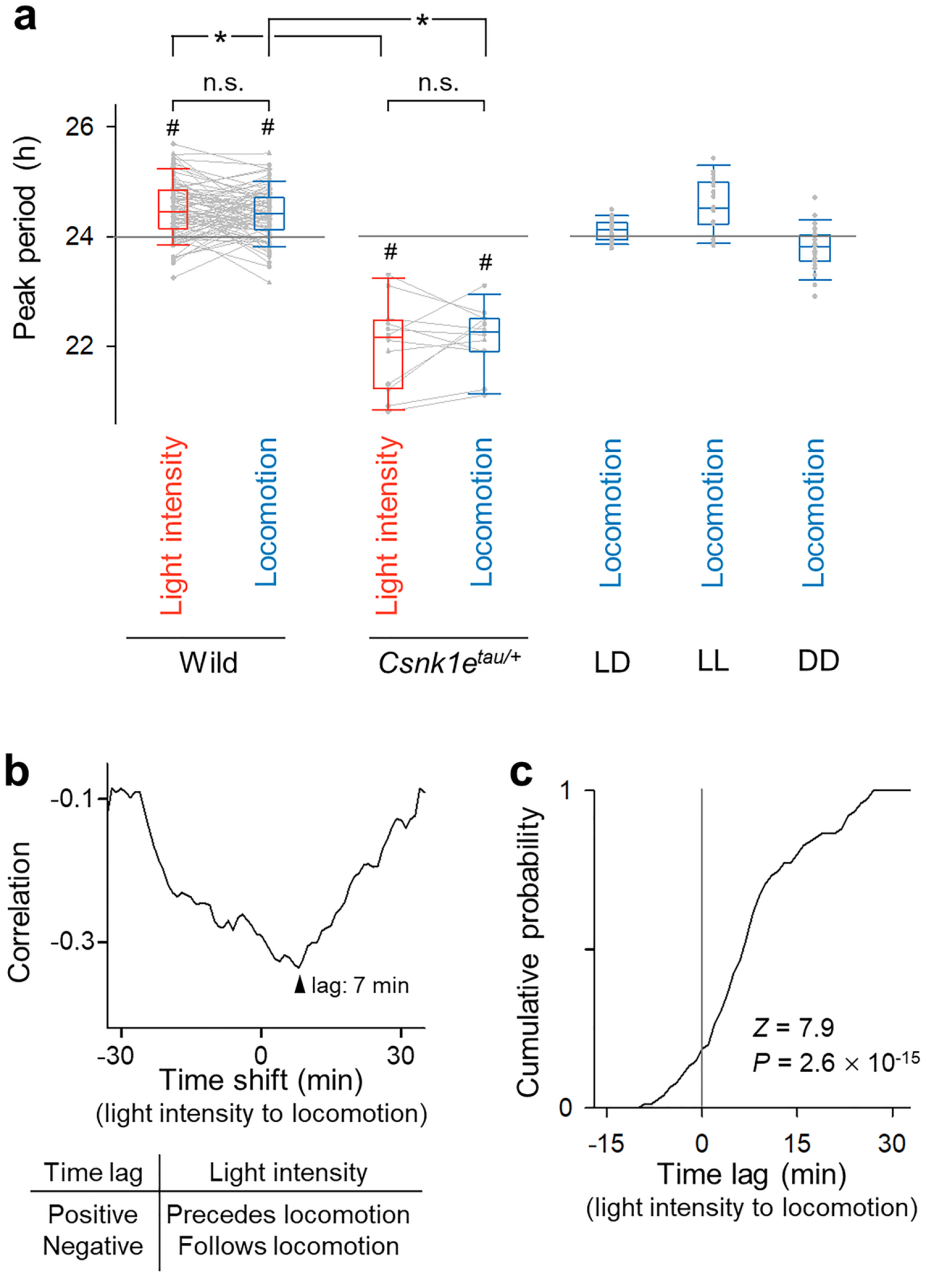
Relationship between self-selected illuminance rhythm and autonomous circadian behavior rhythm. (**a**) Peak periods of illuminance and locomotion in mice that could modulate the chamber lighting. The data were collected from 76 wild-type mice, 12 *Csnk1e*^*tau/*+^ mutant mice (reported to have shorter circadian rhythms), and wild-type mice under 12-h light/12-h dark conditions (LD, 27 mice), continuous light (LL, 24 mice), and continuous dark (DD, 24 mice) conditions. The *gray* lines connect the data collected from the same mice. *P < 0.05, paired *t*-test; ^#^*P* < 0.05, *Z* test *vs.* 24 h. (**b**) Representative cross-correlation between the illuminance and locomotion in a light-modulating mouse. The horizontal axis represents the time shift of the illuminance signal to the locomotion signal, whereas the vertical axis signifies the cross-correlation. Note that mice are more active in the dark and that a time lag exists between illuminance and locomotion. The arrowhead indicates the largest negative correlation between the two signals. The time lag was positive (7 min), indicating that the illuminance change preceded the locomotion change. (**c**) Cumulative probability distribution of the time lag with the largest negative correlation (calculated in (**b**), for example) for all light-modulating mice. The gray vertical line indicates no time lag between the changes in illuminance and locomotion; specifically, the two signals synchronously fluctuate. This graph demonstrates that the mean ± standard deviation (SD) time lag was 8.0 ± 8.8 min, which is significantly greater than zero; thus, changes in illumination preceded changes in locomotion. *n* = 76 mice, *P* = 2.6 × 10^–15^, *Z* = 7.9, Z test *vs.* 0 min.

We analyzed the periodicity of self-controlled illuminance and locomotion using the wavelet transform and found cycles of approximately 24 h in both illuminance and locomotion (Supplementary Fig. 2). Since the powers of both cycles were nearly saturated by Day 3, we calculated both powers of the illuminance and locomotion rhythms after Day 3. Based on the periodogram analysis of a total of 76 mice, we detected circadian periods of 24.5 ± 0.5 h and 24.4 ± 0.5 h in illuminance and locomotion, respectively (Fig. 2a), both of which significantly deviated from 24 h (*P* = 4.6 × 10^–17^, *Z* = 8.4 (for illuminance), *P* = 1.7 × 10^–16^, *Z* = 8.2 (for locomotion), *Z* test *vs.* 24 h) but did not significantly differ in length (*P* = 0.36, *t*_75_ = 0.93, paired *t*-test). When the experiments were extended to 30 d (*vs.* 10 d) in 9 mice, the circadian rhythm remained unchanged (Supplementary Fig. 3; *P* = 0.47, *F*_2,24_ = 0.76 (for illuminance), *P* = 0.62, *F*_2,24_ = 0.48 (for locomotion), one-way ANOVA).

These experiments were repeated using *Csnk1e*^*tau/*+^ and *Csnk1e*^*tau/tau*^ mutant mice, in which their circadian pacemakers were accelerated by the destabilization of the PERIOD proteins^3,4^. The peak cycles of the 12 *Csnk1e*^*tau/*+^ mice were 22.0 ± 0.8 h for illuminance and 22.1 ± 0.5 h for locomotion; both of these cycles were significantly shorter than 24 h (Fig. 2a, Supplementary Fig. 4a; *P* = 1.3 × 10^–17^, *Z* = − 8.5 (for illuminance), *P* = 2.2 × 10^–16^, *Z* = − 8.2 (for locomotion), *n* = 12 mice,* Z* test *vs.* 24 h). We obtained similar results using one *Csnk1e*^tau/tau^ mouse (Supplementary Fig. 4b; 21.4 h (for illuminance); 21.2 h (for locomotion)). Thus, the rhythms in the illuminance and behavior were controlled by internally determined circadian clocks.

### The changes in illuminance precede those in locomotion

We computed the cross-correlation of frequency domains between the illuminance and locomotion. Because mice are more active in the dark, we investigated the negative peaks in the cross-correlations (Fig. 2b). On average, the time lag of the negative peaks was 8.0 ± 8.8 min, which was significantly greater than 0 (Fig. 2c; *P* = 2.6 × 10^–15^, *Z* = 7.9, *n* = 76 mice, *Z* test *vs.* 24 h), indicating that the changes in illuminance preceded the changes in locomotion.

The number of nose pokes into the Up and Down holes was significantly lower than the number of nose pokes into the Blank hole (Supplementary Fig. 1c; *P* = 1.5 × 10^–6^, *t*_75_ = − 5.2 (for Up *vs.* Blank), *P* = 6.2 × 10^–6^, *t*_75_ = − 4.8 (for Down *vs.* Blank), multiple paired *t*-tests with Bonferroni correction). Thus, the mice were more likely to avoid poking the functional holes than the nonfunctional hole. Next, we designed a control experiment in which the holes were randomly assigned to an Up or Down function at each nose poke. Even in this random configuration, the mice exhibited circadian rhythms of illuminance (24.5 ± 0.5 d) and locomotion (24.4 ± 0.4 d) (Supplementary Fig. 5). Overall, the mice actively altered their circadian rhythms by controlling the illuminance, and this environmental rhythm, in turn, regulated their locomotor activity.

We also designed three conventional control groups: (1) the light–dark (LD) group, in which the illuminance was fixed at the typical 12-h light/12-h dark cycle and maintained at level 4 (*i.e.*, 210 lx) during the light period; (2) the light-light (LL) group, in which the illuminance was fixed at level 4 (*i.e.*, 210 lx) continuously over 24 h; and (3) the dark-dark (DD) group, in which the illuminance was fixed at level 1 (*i.e.*, 0.21 lx) continuously over 24 h (Supplementary Fig. 6). The periods of the locomotor activity rhythms were 24.1 ± 0.2 h in the LD group (*n* = 27 mice), 24.6 ± 0.5 h in the LL group (*n* = 24 mice), and 23.8 ± 0.4 h in the DD group (*n* = 24 mice) (Fig. 2a). The mean period of the LL group was similar to that of the light-modulating (LM) group described above (*P* = 0.11, *t*_98_ = − 1.5, Student’s *t*-test). However, the locomotor pattern differed between the two groups; the actograms of the LM group showed a clearer separation between the active and inactive phases, compared to those of the LL group (as seen in a comparison of Fig. 1b with Supplementary Fig. 6b); in the LL group, short active periods occurred many times throughout the day. Furthermore, the circadian oscillation power of locomotion in the LL group (4.3 ± 1.8 a.u.) was significantly weaker than that in the LM group (6.7 ± 2.3 a.u.; *P* = 7.6 × 10^–3^, *t*_98_ = 2.8, Student’s *t*-test). Therefore, we concluded that the circadian rhythm of the LM group was different from that of the conventional LL group.

### The circadian phases of the light-forced mice lag behind those of the light-modulating mice

**Figure 3:**
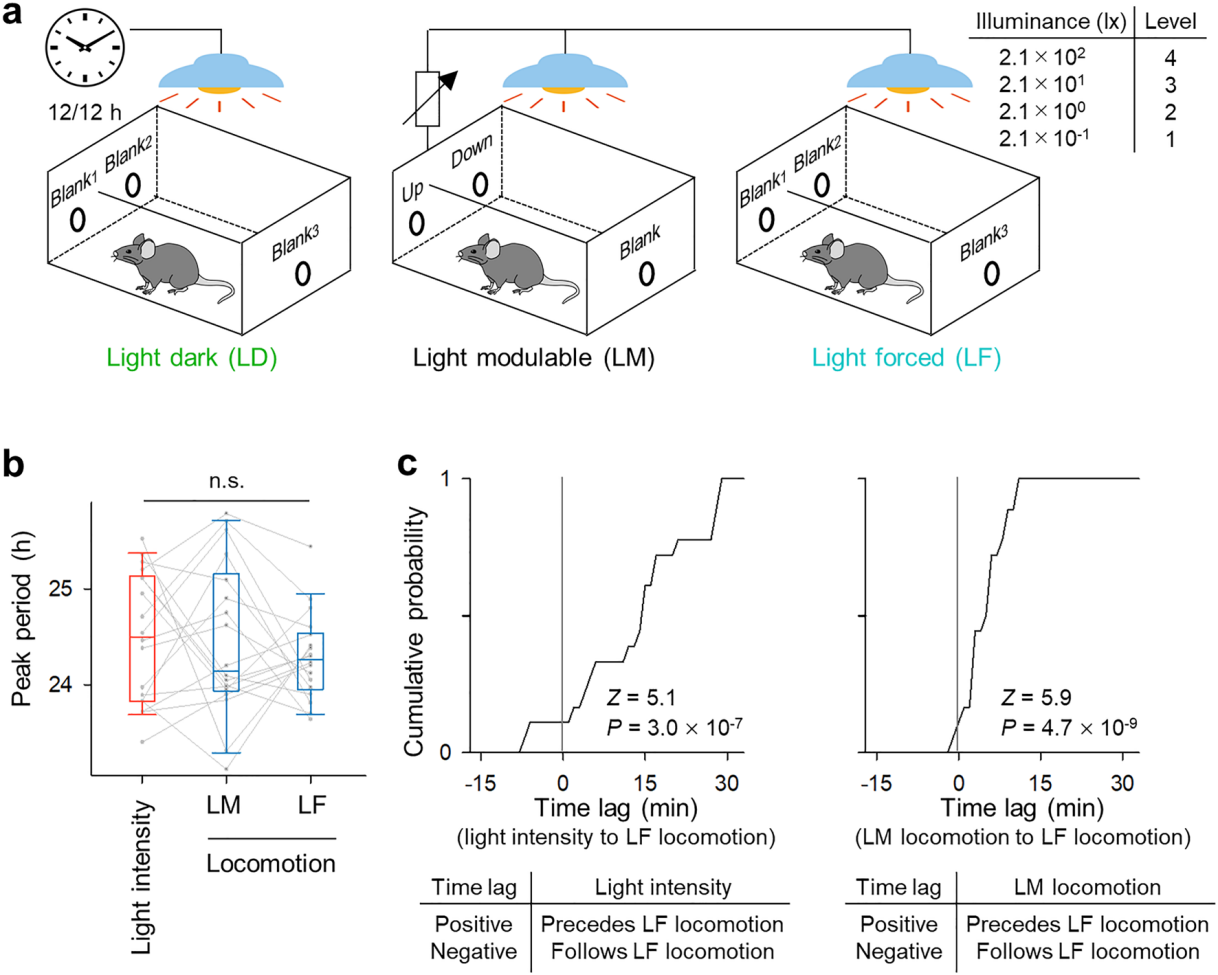
The circadian phases of light-forced mice lag behind those of light-modulating mice. (**a**) Schematic of the experimental chambers used for the light-modulating (LM) group and two control groups. The left panel represents a normal light–dark (LD) paradigm in which the illuminance was set to 12 h light/12 h dark regardless of nose pokes. The middle panel indicates the LM condition in which the illuminance was controlled by mouse nose pokes. The right panel represents the light-forced (LF) condition, a control group in which the illuminance was controlled by the LM mice (i.e., the poke holes were nonfunctional in the LF chambers). (**b**) Peak period of illuminance (left) and locomotion (middle) of the LM and LF groups (right). The data for identical individuals are connected by a gray line. *n* = 18 mice. (**c**) Similar to Fig. 2c, but the cumulative distributions of the time lag between illuminance (determined by the LM group) and locomotion in the LF group (left) and between locomotion in the LM group and locomotion in the LF group (right). The time lag is calculated in the same way as in Fig. 2b, c. The gray vertical line indicates no time lag between two cycles. *n* = 18 mice.

To isolate the effects of the illuminance cycle generated by the LM mice on other mice unable to control the light, we created a new control group, called the light-forced (LF) group. For this group, the illuminance was yoked to that of the LM mice in different chambers; specifically, each LM mouse controlled the illuminance for its own chamber and another chamber housing the LF mouse. Thus, the total light exposure and the timing of the light changes were identical between the LM and LF groups. The experiments were conducted with 18 sets of three mice (*i.e.*, one LD mouse, one LM mouse, and one LF mouse) (Fig. 3a), for a total of 54 mice (*i.e.*, 18 from each condition). In these experiments, we confirmed that the LM group had no preference among the illuminance levels (Supplementary Fig. 7a; *P* = 0.81, χ^2^ = 0.06, *n* = 18 mice, Cochran-Armitage trend test) and that the amount of locomotion was significantly greater during the darker periods in both the LM and LF groups (Supplementary Fig. 7b; *P* = 5.7 × 10^–4^, *t*_17_ = 4.2, *n* = 18 mice, paired *t*-test (for LD), *P* = 9.1 × 10^–6^, *JT* = 546, *Z* = -4.3 *n* = 18 mice, Jonckheere-Terpstra test (for LM), *P* = 2.2 × 10^–3^, *JT* = 689, *Z* = -2.8, *n* = 18 mice, Jonckheere-Terpstra test (for LF)). The LM group exhibited fewer nose pokes into the Up or Down holes than into the Blank holes (*P* = 0.02, *t*_17_ = − 2.4 (for Up or Down *vs.* Blank), multiple paired *t*-tests with Bonferroni correction), whereas neither the LD nor LF groups exhibited any preference among the three Blank holes in their chambers (Supplementary Fig. 7c; *P* = 0.74, *F*_2,51_ = 0.31 (for LD), *P* = 0.71, *F*_2,51_ = 0.34 (for LF), one-way ANOVA).

The period of the locomotor activity rhythm did not significantly differ between the LM and LF groups (Fig. 3b, Supplementary Fig. 8; *P* = 0.41, *t*_17_ = 0.83, multiple paired *t*-tests with Bonferroni correction). However, the time lag between the changes in illuminance and the changes in locomotion in the LF group was 13.6 ± 11.2 min (*n* = 18 mice); this value was significantly larger than 0 min (Fig. 3c; *P* = 3.0 × 10^–7^, *Z* = 5.1, *Z* test *vs.* 0), and the time lag between the locomotion rhythms in the LM and LF groups was 4.7 ± 3.8 min, which was significantly greater than 0 min (Fig. 3c; *P* = 4.7 × 10^–9^, *Z* = 5.8, *Z* test *vs.* 0). Thus, the LF mice exhibited delayed locomotor responses to the changes in illumination and locomotion of the LM mice.

## Discussion

Light is the most prominent daily environmental time cue that entrains the circadian clock. We developed a system in which mice could choose the light intensity of their home cage. In this self-controlled light condition, the mice showed a willingness to adjust the brightness of their environment to their advantage. Although mice are nocturnal and typically prefer dim environments, we unexpectedly found that the mice did not maintain consistently low light levels in their chambers. Instead, they periodically increased their brightness over a period of ~ 24.5 h. Under this self-controlled light cycle, the mice exhibited the same period of locomotor activity rhythms. The illuminance phase preceded the locomotor activity. This behavior is potentially similar to how humans, as diurnal creatures, turn off lights at night to go to sleep.

Previous studies aimed at measuring the period of circadian rhythms in humans have yielded inconsistent results^5,6^. Most human temporal isolation studies have used self-controlled light–dark cycles to measure internal circadian rhythms. The participants controlled room lighting based on their preferences and demonstrated a circadian rhythm of approximately 25 h^6^. Researchers have also determined the human circadian period using the forced desynchrony method. In this method, the onset of melatonin in dim light was determined by a constant or semi-constant routine^5^. Then, the subject typically remained in the laboratory and was forced to sleep and wake with a period outside the range of entrainment (typically, a 28-h cycle), after which the dim light melatonin onset was determined again. The circadian period determined by this method was approximately 24.2 h. A possible explanation for the difference in the periods determined by these two different methods is the effect of the self-controlled light. Our results from the current mouse study concur with the results from previous human studies; however, the mechanism underlying the lengthening of the period by self-controlled illumination remains to be elucidated. Taken together, these results indicate that the interaction between the environmental light and the circadian clock is likely to be more complex than previously believed and should be further investigated. Nevertheless, our study provides a novel approach and tool for future studies of circadian rhythms in mice^7^, rats^8^, and other rodent species^9^.

## Methods

### Animals

The animal experiments were performed with the approval of the Animal Experiment Ethics Committee at the University of Tokyo (approval number: P29-11) and according to the University of Tokyo Guidelines for the Care and Use of Laboratory Animals. These experimental protocols were carried out in accordance with the Fundamental Guidelines for Proper Conduct of Animal Experiment and Related Activities in Academic Research Institutions (Ministry of Education, Culture, Sports, Science and Technology, Notice No. 71 of 2006), the Standards for Breeding and Housing of and Pain Alleviation for Experimental Animals (Ministry of the Environment, Notice No. 88 of 2006), Guidelines on the Method of Animal Disposal (Prime Minister's Office, Notice No. 40 of 1995) and the ARRIVE guidelines. Experiments were performed using 8-week-old or older male littermates of the C57BL/6J mice (Japan SLC, Shizuoka, Japan) and the B6.129-*Csnk1e*^*tm1Asil*^/J mice (Stock No: JR#016183, Jackson Laboratory).

### Genotyping

Genotyping was performed in accordance with a routine procedure established previously^10^. Small tissues obtained from the earlobes of mice were lysed with proteinase K, and genomic DNA was extracted and purified by a spin column method using the DNeasy Blood & Tissue Kit (69504, QIAGEN, Osaka, Japan) according to the manufacturer’s protocol. Genotyping was performed by polymerase chain reaction (PCR) using GoTaq Green Master Mix (M7122, Promega, Madison, WI, USA) and appropriate pairs of the following primers (Eurofins Genomics, Tokyo, Japan): forward, 5′-CAC CTG GGC ATT GGT GAG T-3′; reverse, 5′-GGA GGT CAA GGG GCC AGT-3′. The PCR parameters were as follows: 94 °C for 2 min, 10 cycles of [94 °C for 20 s and 64 °C for 30 s] with a 0.5 °C decrease each cycle, then 28 cycles of [94 °C for 10 s, 60 °C for 15 s and 72 °C for 10 s]. The PCR products and a DNA ladder (NE-MWD 100P, NIPPON Genetics, Tokyo, Japan) were analyzed by electrophoresis through 3% agarose gel containing nucleic acid stain and Midori Green Advance (NE-MG04, NIPPON Genetics) and then imaged using a gel documentation system (AE6914, ATTO, Tokyo, Japan).

### Apparatus

The experiments were performed in 12 experimental boxes (300 mm wide × 400 mm long × 395 mm tall; made of vinyl chloride; OP-3802De, O’Hara, Tokyo, Japan), and each was placed inside a soundproof box (500 mm wide × 600 mm long × 500 mm tall; BrainScience-idea, Osaka, Japan), in a windowless room on the basement floor^11,12^. An LED panel (maximum illuminance, 1,500 lx; color temperature, 6000 K) was placed on the top of each experimental box. The illuminance was regulated with pulse-width modulation using an Arduino Mega 2560 microcomputer board (ATmega328, Arduino, NY, USA). An infrared camera (Ailipu Technology, Guangdong, China) was placed in the experimental box to record the animal behavior.

### Experimental conditions

The mice were randomly divided into the LM, LF and LD groups. Unless otherwise specified, their free-moving behavior was recorded for 10 d using an overhead infrared camera (recording at 3 fps). The mice assigned to the LM group were housed in boxes with three nose-poke holes on the wall (Fig. 1A). When the mice poked their noses into the Up hole, the illuminance was increased by one level in a logarithmic series (*i.e.*, 0.21 lx (level 1), 2.1 lx (level 2), 21 lx (level 3), or 210 lx (level 4)); note that physiological visual sensitivity varies on a logarithmic scale and that the number of steps, 4, was chosen because 2 bits (= 4) is convenient for computer control. When the mice poked their noses into the Down hole, the illuminance decreased by one level. The mouse nose pokes into the Blank hole did not trigger any change in the illuminance. The nose pokes were detected by infrared sensors placed inside the holes, and the time stamps were recorded using an Arduino instrument. The LF mice were housed in the same type of chamber but had no control over the illuminance, which was yoked to that of the LM group. The LD mice were housed in the same type of chamber with the illuminance automatically set to switch between dark (level 1) and light (level 4) conditions every 12 h. In this room, all poke holes were nonfunctional (*i.e.*, Blank holes); thus, no change in illumination occurred upon the mouse nose pokes. The mice in each group were reassigned to one of the other groups after 10 d; thus, every mouse experienced all conditions (LD, LM, and LF) once during an experimental period of 30 d. For all experimental groups, food and water were freely available. The floors of the chambers were filled with a sufficient amount of animal bedding in advance, reducing the number of bedding replacements needed since these replacements would interrupt the experiments and provide cues about the environment outside of the chamber. The experimental chambers had a drawer at the bottom, enabling quick replacement of the animal bedding. The environment inside the soundproof box was kept constant at a temperature of 22 °C and a humidity of 50%.

### Data analysis

The data were analyzed using MATLAB (MathWorks, Natick, MA, USA) and Python (Python Software Foundation). The summarized data are displayed as box-and-whisker plots. Representative values are reported as the mean ± standard deviation (SD) unless otherwise specified. *P* < 0.05 was considered to indicate statistical significance. When multiple pairwise comparisons were performed, we applied Bonferroni correction to the original *P* values and compared the corrected *P* values with 0.05.

The animals’ moment-to-moment positions were tracked using DeepLabCut, a markerless tracking system^13,14^. Single-plot actograms provided a visualization of one-minute changes in locomotion and illuminance. A wavelet transform was applied to detect the power of each frequency (0.5–5 cycles per d) for 1-min windows^15–19^. The peak period was defined as the period with the highest total power after Day 2.

The two rhythms of illuminance and locomotion (Fig. 1b) were circularly shifted to calculate the cross-correlation function C(τ) (− 30 min < τ < 30 min) between the two cycles (Fig. 2b)^15,16,20–22^. The time lag was defined as the amount of time shift (τ_max_), where C(τ_max_) was the minimum value (Fig. 2b, c). A positive value of the time lag represented the change in illuminance preceding the change in locomotion (Fig. 2c). Similarly, the cross-correlation function and time lag were calculated for the pairs of (1) illuminance and locomotion in the LF group (Fig. 3c, left) and (2) locomotion in the LM and LF groups (Fig. 3c, right).

### Supplementary Information

Supplementary Information 1.

Supplementary Video 1.

## Data Availability

All data used in this study are available from the corresponding authors upon reasonable request. Source data are provided within this paper.
