# Supplementary material for: Lengthened circadian rhythms in mice with self-controlled ambient light intensity
Source: Sci Rep. 2024 Apr 2;14:7778. doi: 10.1038/s41598-024-58415-x (PMC10987682; doi:10.1038/s41598-024-58415-x)
Supplement: Supplementary file 1 — Supplementary Information 1. [file 41598_2024_58415_MOESM1_ESM.pdf]

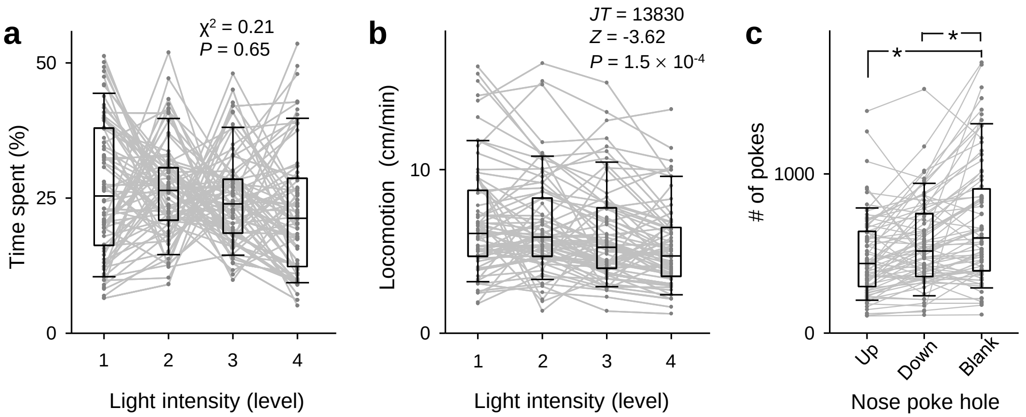

Supplementary Fig. 1. Increased illuminance reduces locomotion.
a, Proportions of the total time spent at the four illuminance levels. The *gray* lines connect data from the same individual. The proportions of time at the four illuminance levels did not significantly differ. $P = 0.65$, $\chi^2_{225} = 0.21$, χ^2 test, $n = 76$ mice. Note that the light intensity levels 1, 2, 3, and 4 correspond to 0.21, 2.1, 21, and 210 lx, respectively. **b**, Mean locomotion per minute. Locomotion was negatively correlated with illuminance. $P = 1.5 \times 10^{-4}$, $JT = 13{,}830$, $Z = -3.62$, Jonckheere-Terpstra test, $n = 76$ mice. Note that light intensity levels 1, 2, 3, and 4 correspond to 0.21, 2.1, 21, and 210 lx, respectively. **c**, Total number of nose pokes into the Up, Down, and Blank holes. The number of nose pokes into the Blank hole was significantly greater than that into the other (functional) holes. $^*P < 0.05$, paired *t*-test with Bonferroni correction, $n = 76$ mice.

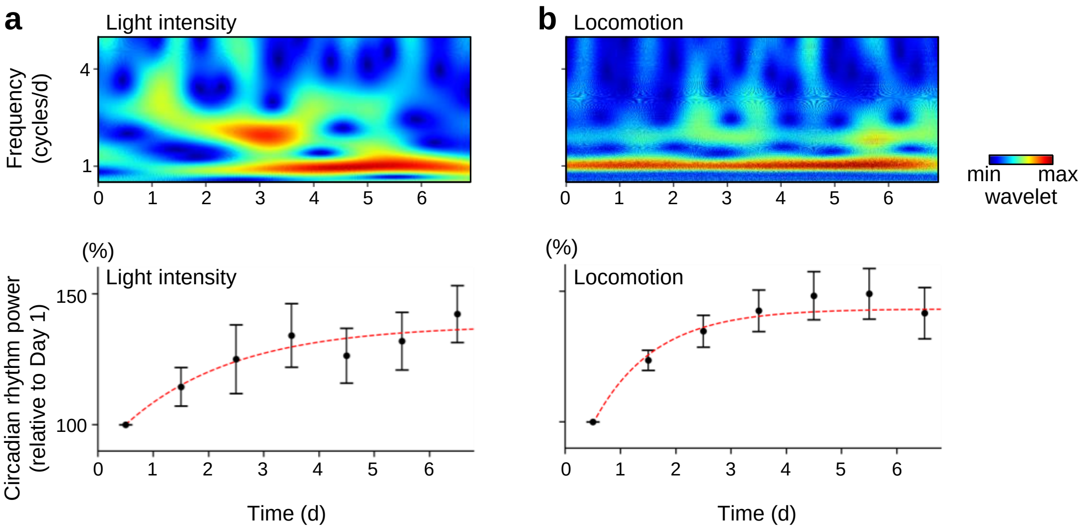

Supplementary Fig. 2. Circadian rhythms under light-modulable conditions are formed within 3 d. **a**, *Top*: Representative power spectrogram of illuminance during the first 7 d. *Bottom*: Time course of the power of the circadian rhythm (*i.e.*, the mean power between 22.5–25.5 h). The data represent the mean ± the standard error of the mean (SEM) with a quadratic curve fit using the least-squares method (*red*). **b**, Same as **a**, but for locomotion.

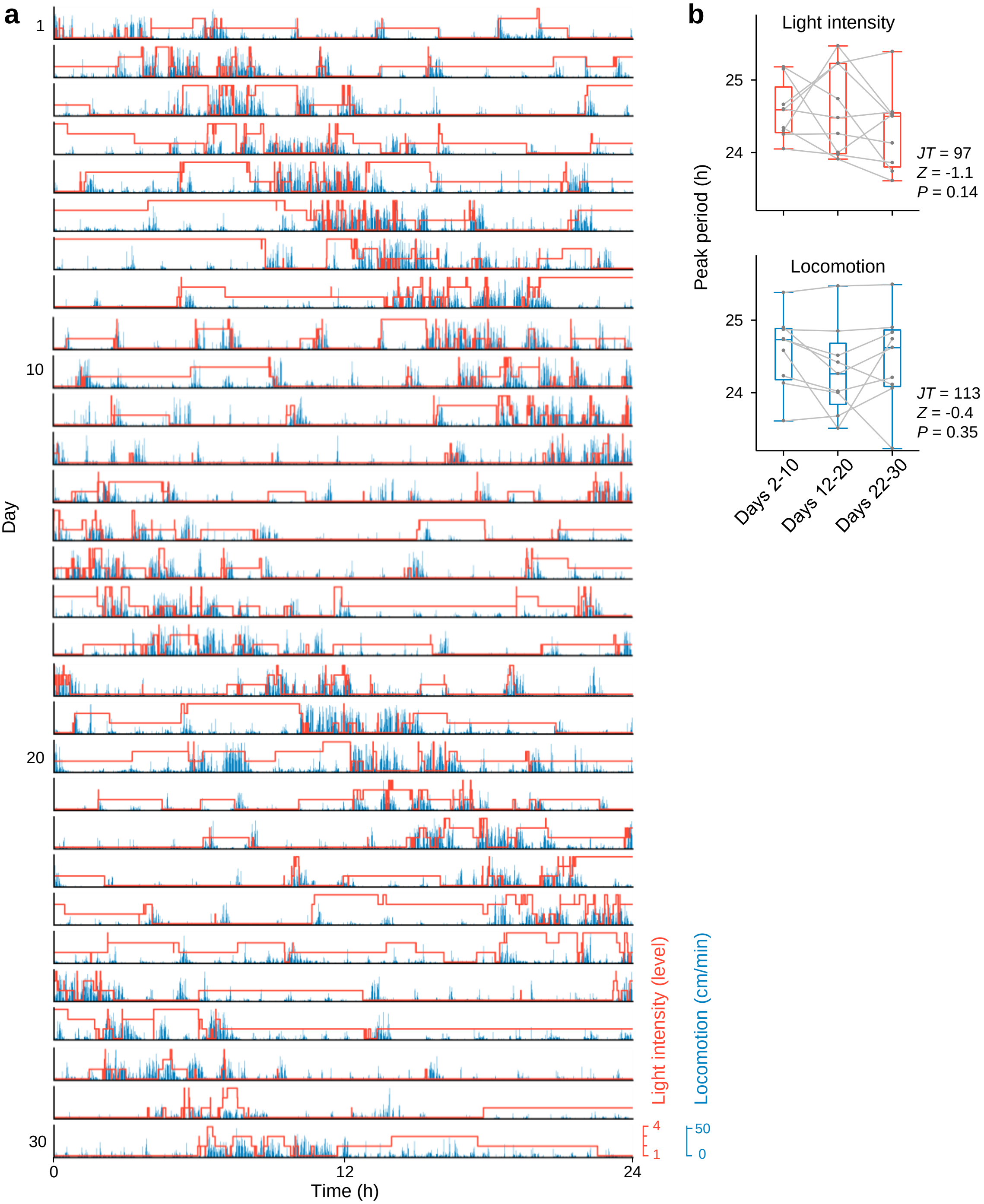

Supplementary Fig. 3. Prolonged circadian rhythms in self-selected illuminance modulation and locomotion persist for at least 30 d.

a, Representative data on illuminance (*red*) and locomotion (*blue*) from an LM mouse over 30 d. The horizontal axis represents time, and each row represents a single day. **b**, The peak periods (collected from 9 mice) of illuminance (*top*) and locomotion (*bottom*) plotted for Days 2–10, 12–20, and 22–30. Peak periods from the same mouse are connected by a *gray* line. The peak periods did not significantly change over time. Jonckheere-Terpstra test, $n = 9$ mice.

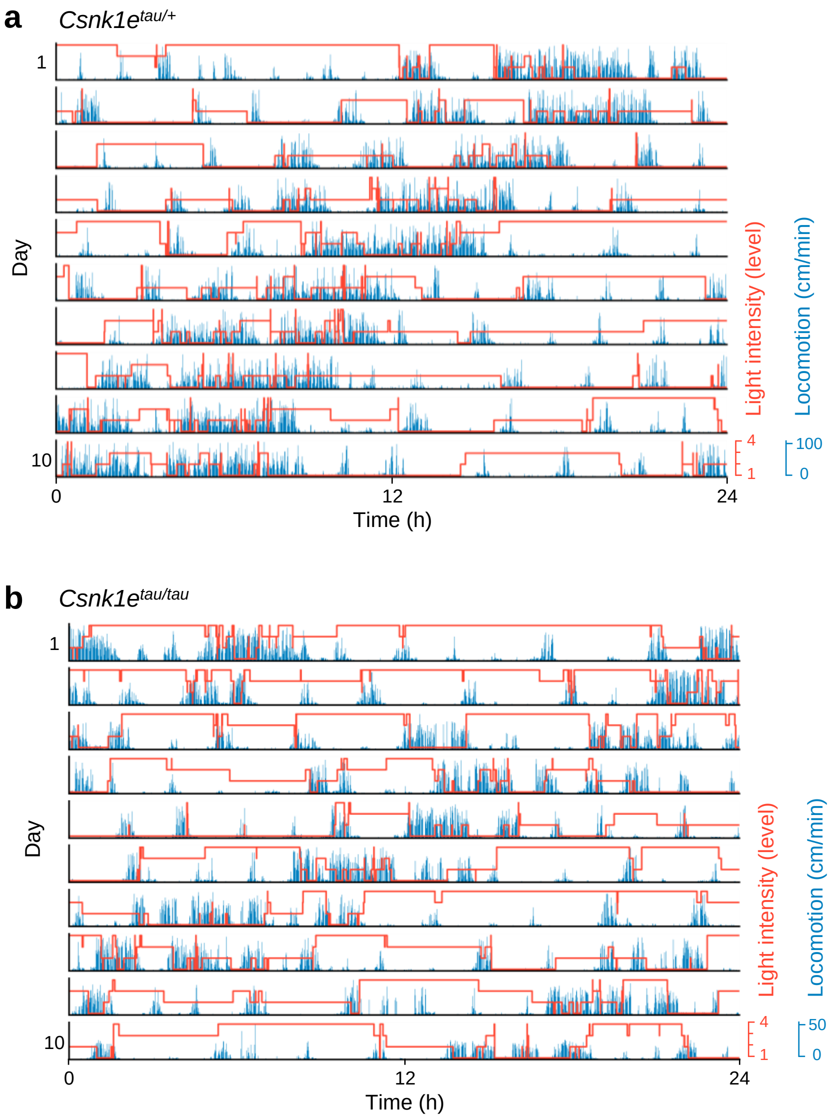

Supplementary Fig. 4. *Csnk1e* mutant mice exhibit shorter circadian rhythms under light-modulable conditions.

a, Representative actograms from a heterozygous *Csnk1e*$^{tau/+}$ mouse, a strain known to have a circadian rhythm of 21.8 ± 0.05 h. **b**, Same as **a**, but from a homozygous *Csnk1e*$^{tau/tau}$ mouse, a strain known to have a circadian rhythm of 20.0 ± 0.07 h.

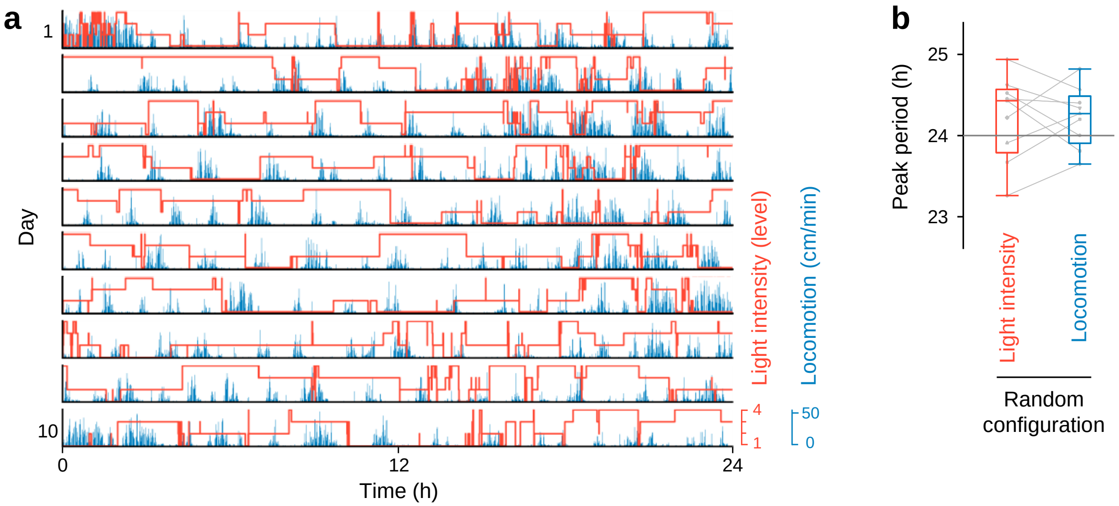

Supplementary Fig. 5. Mice exhibit prolonged circadian rhythms even in a chamber in which the nose-poke holes for light modulation are randomized.

a, Representative actogram of illuminance (*red*) and locomotion (*blue*) of a mouse in a chamber in which the Up and Down functions of the two holes are randomly allocated at each nose poke (called the random configuration). **b**, Peak cycles of illuminance and locomotion of 9 mice under the random configuration.

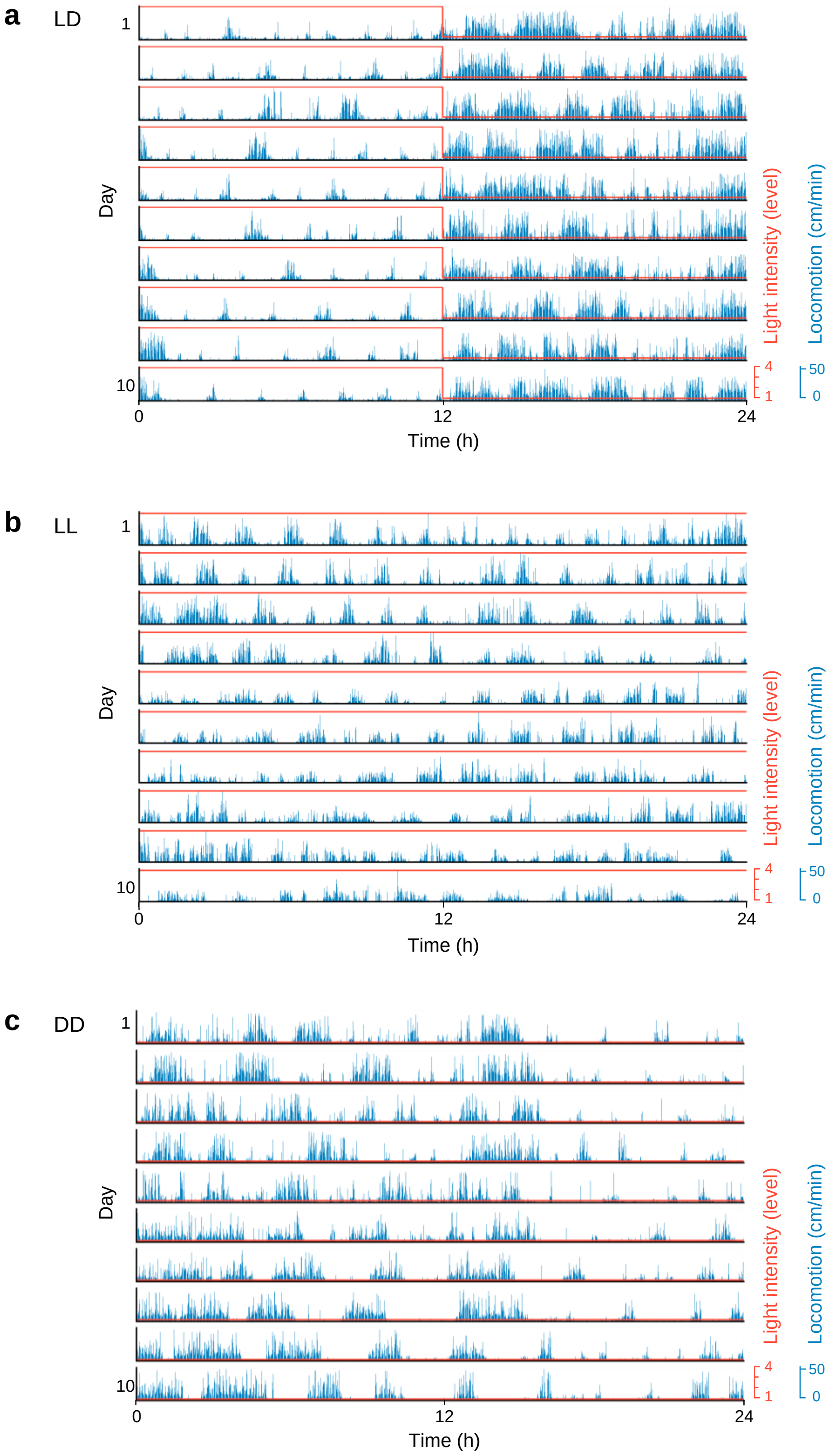

Supplementary Fig. 6. Mice exhibit characteristic circadian rhythms under different light–dark conditions.

a-c, Representative actograms of mice under 12-h light/12-h dark (LD, **a**), continuous light (LL, **b**), and continuous dark (DD, **c**) conditions.

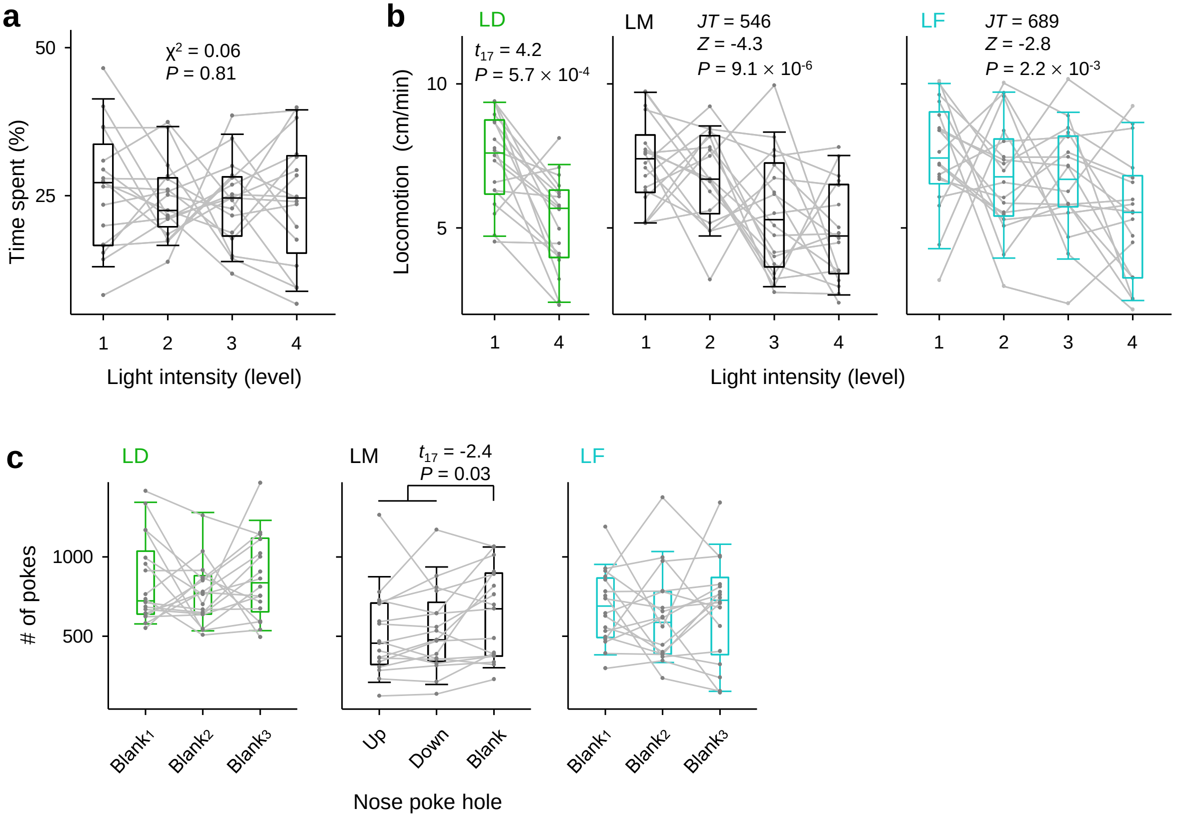

Supplementary Fig. 7. Higher illuminance reduces locomotion.

a, Percentages of the time at the four illuminance levels in the LM and LF groups. The *gray* lines connect the data of the same individual. Note that the LM and LF groups were paired and thus exposed to the same illuminance levels. The percentages of time at each illuminance level did not significantly differ. P = 0.81, χ^2 = 0.06, Cochran-Armitage test, n = 18 mice. **b**, Locomotion at each illuminance level in the LD (*left*), LM (*middle*), and LF (*right*) groups; mice were more active under darker conditions. $P = 5.7 \times 10^{-4}$, t_{17} = 4.2, paired t-test for 18 LD mice; $P = 9.1 \times 10^{-6}$, JT = 546, Z = -4.3, Jonckheere-Terpstra test for 18 LM mice; $P = 2.2 \times 10^{-3}$, JT = 689, Z = -2.8, Jonckheere-Terpstra test for 18 LF mice. **c**, Total number of nose pokes into each hole in each group. Each *gray* line signifies a single mouse. The number of nose pokes into the Up or Down hole was significantly lower than that into the Blank hole in the LM group. P = 0.03, t_{17} = -2.4, paired t-test, n = 18 mice. In the LD and LF groups, no significant difference was observed in the number of nose pokes into these holes. LD: P = 0.74, $F_{2,51}$ = 0.31; LF: P = 0.71, $F_{2,51}$ = 0.34, one-way ANOVA, n = 18 mice each.

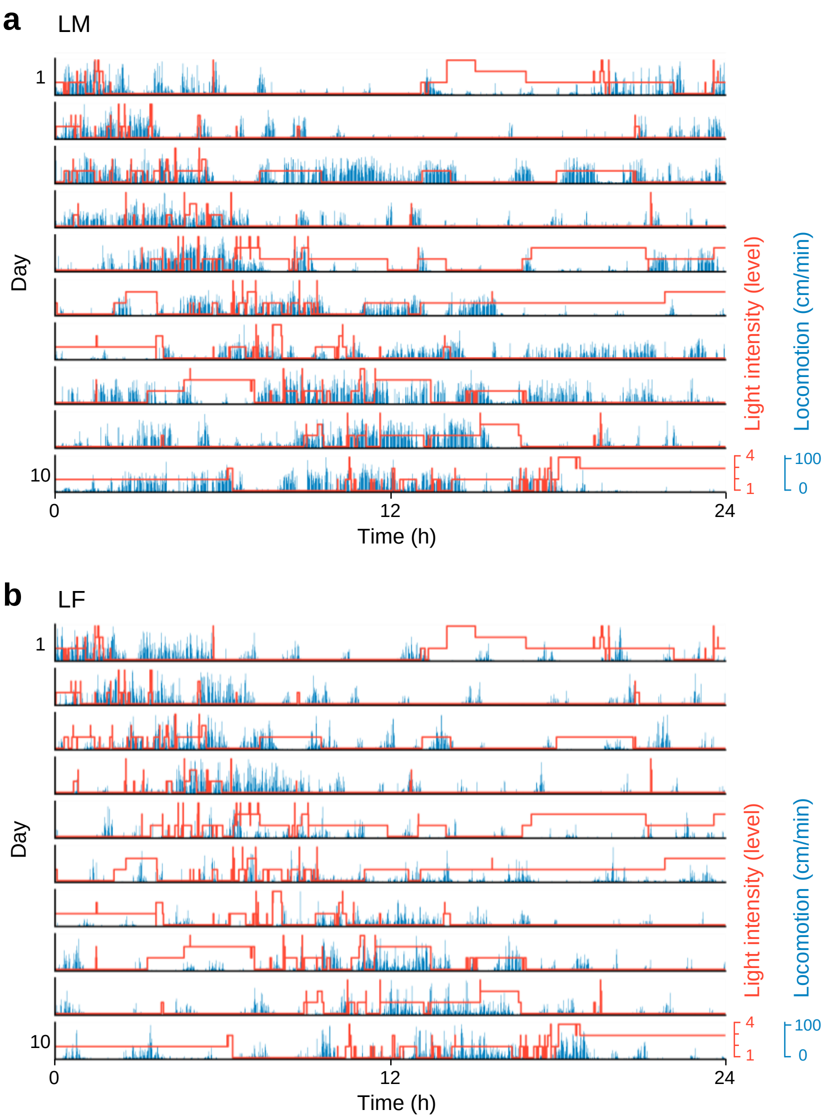

Supplementary Fig. 8. Both LM and LF mice exhibit prolonged circadian rhythms. Representative actograms of an LM (**a**) mouse and a yoked LF mouse (**b**), both of which shared the same illuminance levels.

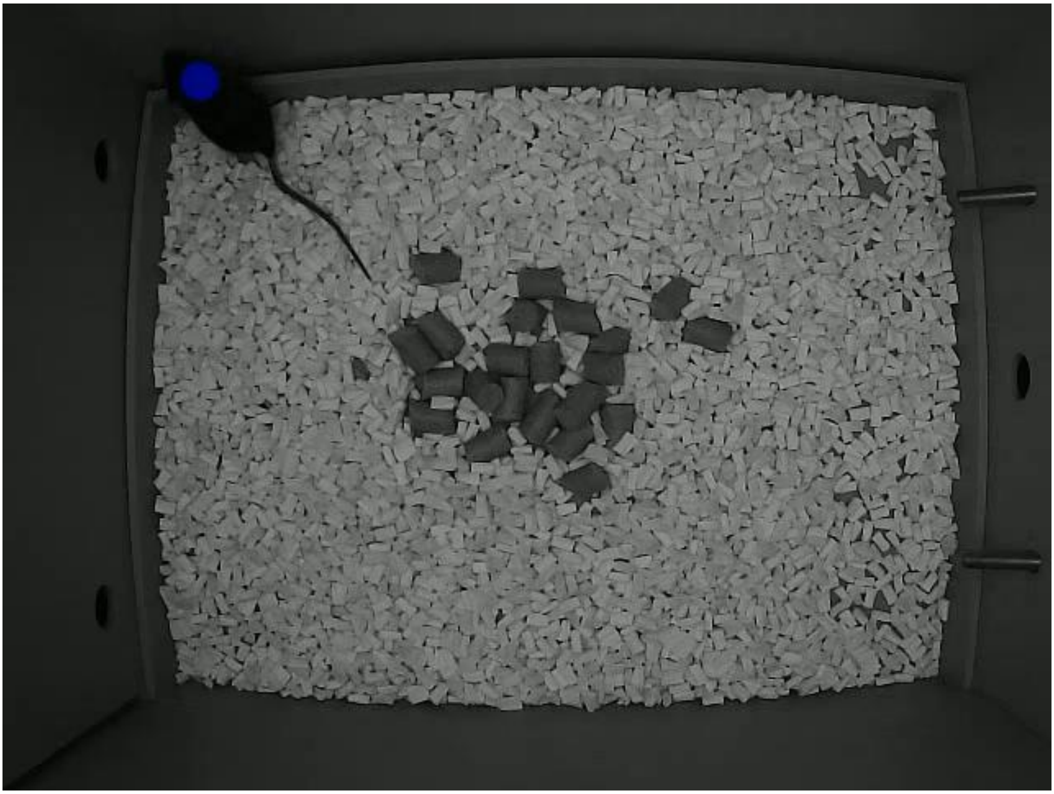

Supplementary Movie.
A light-modulating (LM) mouse pokes its nose into the Up hole (*top, left*) or the Down hole (*bottom, left*) to change the illuminance of the chamber. To capture animal movement, the brightness of the image was automatically adjusted by changing the sensitivity and exposure of the camera.
